# The Clinical Implications of Nocebo Effects for Biosimilar Therapy

**DOI:** 10.3389/fphar.2019.01372

**Published:** 2019-11-29

**Authors:** Luana Colloca, Remo Panaccione, T. Kevin Murphy

**Affiliations:** ^1^Department of Pain Translational Symptom Science, School of Nursing, University of Maryland, Baltimore, MD, United States; ^2^Department of Anesthesiology/Psychiatry, School of Medicine, University of Maryland, Baltimore, MD, United States; ^3^IBD Unit, Division of Gastroenterology and Hepatology, Department of Medicine, University of Calgary, Calgary, Canada; ^4^Pfizer Inc, New York, NY, United States

**Keywords:** biosimilar, nocebo effect, patient’s expectancies, patient–clinician communication, placebo effect

## Abstract

Nocebo effects encompass negative responses to inert interventions in the research setting and negative outcomes with active treatments in the clinical research or practice settings, including new or worsening symptoms and adverse events, stemming from patients’ negative expectations and not the pharmacologic action of the treatment itself. Numerous personality, psychosocial, neurobiological, and contextual/environmental factors contribute to the development of nocebo effects, which can impair quality of life and reduce adherence to treatment. Biologics are effective agents widely used in autoimmune disease, but their high cost may limit access for patients. Biosimilar products have gained regulatory approval based on quality, safety, and efficacy comparable to that of originator biologics in rigorous study programs. In this review, we identified gaps in patients’ and healthcare professionals’ awareness, understanding, and perceptions of biosimilars that may result in negative expectations and nocebo effects, and may diminish their acceptance and clinical benefits. We also examined features of nocebo effects with biosimilar treatment that inform research and clinical practices. Namely, when biosimilars are introduced to patients as possible treatment options, we recommend adoption of nocebo-reducing strategies to avoid negative expectations, including delivery of balanced information on risk–benefit profiles, framing information to focus on positive attributes, and promoting shared decision-making processes along with patient empowerment. Healthcare professionals confident in their knowledge of biosimilars and aware of bias-inducing factors may help reduce the risk of nocebo effects and improve patients’ adherence in proposing biosimilars as treatment for autoimmune diseases such as rheumatoid arthritis and inflammatory bowel disease.

## Introduction

The concept of a nocebo, developed as the negative equivalent of a placebo, has started to draw considerable attention in the clinical research and practice settings (Kennedy, 1961; Pouillon et al., 2018). Nocebo effects are psychological, physiological, and neurobiological phenomena associated with actual or perceived harm that occur as a consequence of patients’ negative expectancies, psychosocial context, and therapeutic environment, not the known pharmacologic actions of treatment (Colloca and Miller, 2011; Colloca and Finniss, 2012; Hauser et al., 2012; Chavarria et al., 2017). Although not as extensively studied as placebo effects, nocebo effects are widely recognized as no less significant, as they are capable of causing patients harm and interfering with the effects of medical treatment (Chavarria et al., 2017; Dodd et al., 2017). Nocebo effects include negative responses to inert interventions administered in laboratory or clinical research (e.g., patients in randomized clinical trials receiving placebo, not active medication, are known to discontinue treatment as a result of adverse events) (Barsky et al., 2002). However, nocebo effects also encompass negative responses, or underlie the absence of positive responses, to active interventions in clinical trials or practice that cannot be explained by the medication’s pharmacologic properties (Colloca and Miller, 2011). Notably, the informed consent process required by clinical research protocols may prompt patients to expect adverse events with treatment, resulting in nocebo adverse events and decreased adherence (Colloca and Miller, 2011; Faasse and Petrie, 2013). Patients’ negative expectations about treatment have also been shown to critically influence its efficacy in analgesic studies (Bingel et al., 2011). Although most studies of nocebo effects have been conducted in the field of pain, these effects have also been demonstrated in other conditions, such as fatigue, gastrointestinal disorders, allergy, and itch (Levine et al., 2006; de la Cruz et al., 2010; Bavbek et al., 2015; Elsenbruch and Enck, 2015; Napadow et al., 2015; Quinn et al., 2017).

The introduction of biosimilars for the treatment of chronic immune-mediated inflammatory diseases initiated a new area of nocebo research. Innovative biologic therapies are widely used in autoimmune diseases such as rheumatoid arthritis and inflammatory bowel disease (IBD) because of their effectiveness (Emery et al., 2013; Akobeng et al., 2014; Nam et al., 2017), but the high cost of these therapies poses an economic burden for healthcare systems and restricts access for many patients (Al Maini et al., 2015; Baumgart et al., 2019). Over the past decade, as patent protection and market exclusivity of biologics such as tumor necrosis factor (TNF) inhibitors have expired, biosimilar products with a high degree of similarity to originator biologics have been approved as therapeutic options. Because biologic agents are created using highly specialized and proprietary processes in living cells, biosimilars are not identical to originator biologics. However, global guidelines established by the World Health Organization require biosimilar authorization to be based on outcomes of rigorous comparability exercises that demonstrate biosimilarity, including quality, non-clinical, and clinical studies (World Health Organization and Expert Committee on Biological Standardization, 2009). Moreover, regulatory pathways established by agencies such as the European Medicines Agency (EMA) and US Food and Drug Administration (FDA) protect against clinically meaningful differences between biosimilars and originators.

The introduction of biosimilars has the potential to provide substantial cost savings for healthcare systems worldwide and to expand treatment choices for patients and clinicians (Dorner et al., 2016; Danese et al., 2017; Mulcahy et al., 2018). From 2014 to 2017, in the United Kingdom (UK), use of biosimilars of the TNF inhibitor infliximab and etanercept was associated with £39 million in cumulative cost savings in rheumatology alone (Aladul et al., 2017). Moreover, in 2018, the UK National Health Service indicated that its planned use of biosimilars of the TNF inhibitor adalimumab is anticipated to save approximately £300 million of the current £400 million spent annually on adalimumab (Davio, 2018). In the US, from 2017 to 2026, biosimilars are expected to reduce direct spending on biologics by approximately $54 billion (Mulcahy et al., 2018). Despite accumulating medical literature that supports biosimilar use and growing recognition of their value (Jacobs et al., 2016a; European Medicine Agency and European Commission, 2017; Jorgensen et al., 2017; US Food and Drug Administration, 2018), several obstacles to more widespread adoption of these agents remain. Slow uptake of biosimilars may reflect gaps in patients’ and clinicians’ knowledge and understanding of these agents’ risks and benefits, and has stimulated interest in the potential role of nocebo phenomena (Rezk and Pieper, 2017; Boone et al., 2018; Germain et al., 2018; Kravvariti et al., 2018; Kristensen et al., 2018; Odinet et al., 2018; Pouillon et al., 2018; Rezk and Pieper, 2018; Scherlinger et al., 2018; Tweehuysen et al., 2018).

Regulatory authorization of biosimilars is typically based on evidence from rigorously conducted clinical studies (e.g., double-blind, equivalence studies) that supports the comparability of biosimilars and originator products. In a recent review of 90 published studies, including both randomized clinical trials and real-world evidence, no changes were observed in safety or efficacy after patients switched from originator biologic to biosimilar medications (Cohen et al., 2018). However, some researchers examining outcomes in patients switched from originator products to biosimilars have observed higher drug discontinuation rates in open-label or real-world studies relative to blinded trials, raising suspicions of possible nocebo effects (Glintborg et al., 2017b; Odinet et al., 2018). Low levels of awareness and misconceptions of biosimilar safety and efficacy may prompt uncertainty and negative attitudes, and could influence treatment adherence and outcomes (Jacobs et al., 2016b; Cohen et al., 2017; Peyrin-Biroulet et al., 2017; Rezk and Pieper, 2017). Although some regulatory authorities have published guidelines on biosimilars to help educate healthcare professionals and patients, such initiatives do not adequately address the potential for nocebo effects or how such effects could be mitigated. In addition, health authority and health insurance company programs and policies have been initiated in Europe and other regions to enhance biosimilar uptake, but these also have not adequately addressed concerns regarding nocebo phenomena (Moorkens et al., 2017; Vogler and Schneider, 2017; Smeeding et al., 2019). The objectives of this narrative ‘scoping’ review are: 1) to present the mechanisms underlying the development of nocebo effects with biosimilar therapy and predictive factors; 2) to explore the possible triggers for, and impact of, nocebo effects when biosimilar medicines are implemented; and 3) to discuss strategies that may mitigate these effects with biosimilars in clinical practice, including educational initiatives and tailored patient–clinician communication and interaction.

## Methods

A qualitative, scoping approach was taken in developing this review (not a quantitative, systematic approach). Our aim was to map the available literature on nocebo effects with biosimilars, a body of literature that had not yet been comprehensively reviewed, using the scoping approach to synthesizing research evidence (Pham et al., 2014). Although a systematic literature review was not performed, a literature search of PubMed was conducted for articles on nocebo effects and biosimilar therapy published between January 2015 and July 2018. Search terms included: biosimilar, clinical, inflammatory, nocebo, placebo effect, rheumatology, and treatment. Reviews, randomized controlled trials, and observational studies were identified. Reference lists from articles identified in this literature search were reviewed and additional publications retrieved if considered relevant to this review. Articles with pertinent information on nocebo effects with biosimilar therapies in clinical research or practice were selected for qualitative synthesis. In addition, pioneering studies of nocebo effects conducted in fields other than rheumatic disease (e.g., pain) are also cited (based on author research/expertise) to improve understanding of the concept of nocebo effects and provide a broader context for research more recently conducted with biosimilars. This article is based on previously conducted studies and does not contain information about any studies with human participants or animals performed by any of the authors.

## Basic Mechanistic Knowledge Informing Clinical Research

The efficacy and tolerability of active treatments such as biosimilars can be jeopardized by nocebo effects and their underlying psychological and neurobiological mechanisms, in addition to myriad contextual and individual factors (Colloca and Miller, 2011; Blasini et al., 2017; Dodd et al., 2017; Evers et al., 2018). From a psychological standpoint, the basic mechanisms contributing to the development of negative expectations and nocebo responses are prior experience of negative therapeutic outcomes (i.e., conditioning) and anticipation of negative outcomes (Colloca and Miller, 2011; Blasini et al., 2017). In individuals who experience a pharmacologic conditioned response, previously administered active treatment elicits reactions imprinted in memory that may help shape future expectations and responses to placebo or active treatments (Colloca et al., 2010; Dodd et al., 2017). Previous negative experiences with pharmacologic treatment of short or long duration can increase the likelihood of harmful effects and reduce the likelihood of therapeutic benefits in the future (Colloca et al., 2010).

Expectancies that influence therapeutic response can also be generated by pre-existing beliefs triggered by verbal information, social observation, or other information sources (Kirsch et al., 2014; Okusogu and Colloca, 2019). In a systematic review of risk factors involved in the development of nocebo effects, the strongest factors were verbal suggestions that treatment exposure triggers symptoms, observation of others experiencing symptoms with treatment, and higher expectations of symptoms (Webster et al., 2016). Verbal suggestion with and without first-hand prior experience can promote nocebo effects on physical symptoms (Flaten et al., 1999; Colloca et al., 2008; van Laarhoven et al., 2011; Bartels et al., 2014; Carlino et al., 2015; Quinn and Colagiuri, 2016). Nocebo effects may also be induced by exposure to information disseminated *via* media outlets, including medical information derived from the internet or posted on social media, advertisements for pharmacologic treatments, and descriptions/warnings about health-related conditions on television or in print (Faasse et al., 2009; Blasini et al., 2017). In addition, individuals may demonstrate behavioral changes after observing others’ behavior, which provides information about specific situations and the consequences of specific actions, without experiencing them first hand (Blasini et al., 2017).

Interestingly, negative expectations underlying nocebo phenomena have been shown to alter activity in certain regions of the brain. For example, in a study of the analgesic efficacy of a potent opioid, Bingel et al. found that negative treatment expectancy in healthy volunteers experiencing constant heat pain abolished the opioid’s analgesic effects (Bingel et al., 2011). Using brain imaging, the investigators showed that these subjective effects were accompanied by significant changes in neural activity in the hippocampus, suggesting that expectancy influences regulatory brain mechanisms.

Although all patients may be susceptible to nocebo effects, certain subgroups may be at particular risk, including women and individuals with psychological disorders such as anxiety (Klosterhalfen et al., 2009; Wells and Kaptchuk, 2012; Data-Franco and Berk, 2013; Corsi et al., 2016; Corsi and Colloca, 2017; Vambheim and Flaten, 2017). The interaction of these factors may explain large variations in nocebo effects seen among individuals (Corsi and Colloca, 2017). In a systematic review, Vambheim et al. found that nocebo responses were more common in women than men, a difference that may stem from higher levels of stress and anxiety in women (Vambheim and Flaten, 2017). However, in a more recent study of the impact of learning on nocebo, a significant relationship was observed between anxiety and nocebo responses regardless of sex (Corsi and Colloca, 2017). A meta-analysis of nocebo effects in the treatment of major depression showed that patients receiving placebo were more likely to report adverse events in phase II clinical trials than in phase III or IV trials, potentially because concerns or uncertainties about antidepressant treatment efficacy elicited nocebo responses in early stage trials (i.e., before efficacy had been established) (Dodd et al., 2015). Pessimists have also exhibited a greater probability of following negative expectations than optimists when given placebos and told that the pills would have unpleasant effects (Geers et al., 2005). Finally, a sense of involvement or control regarding treatment decisions may also influence nocebo effects, as individuals who are not allowed a choice of medications have reported significantly more adverse events than those allowed such a choice (Bartley et al., 2016).

## Nocebo Phenomena and Biosimilar Therapy: Knowledge Gaps, Misperceptions, and Negative Expectations

The potential for nocebo effects to occur in patients with autoimmune disease when switching from originator biologics to biosimilars is a rapidly growing field of study (Boone et al., 2018; Germain et al., 2018; Kravvariti et al., 2018; Kristensen et al., 2018; Odinet et al., 2018; Tweehuysen et al., 2018). The first biosimilar agent (somatropin; Sandoz International GmbH, Holzkirchen, Germany) was approved more than a decade ago, and nearly 50 additional biosimilars have been authorized for use in Europe and the US in the intervening years (Harston and Storaska, 2018). Substantial gaps in patients’ and clinicians’ awareness and understanding of biosimilars, as well as misperceptions about these agents, have nevertheless been identified, which may contribute to uncertainty and negative attitudes towards these innovative therapies and affect their use in clinical practice (Jacobs et al., 2016b; Cohen et al., 2017; Peyrin-Biroulet et al., 2017; Rezk and Pieper, 2017). In an international survey of patients in the US and the European Union, over two-thirds of approximately 3000 respondents overall and about half of those recently diagnosed with a chronic autoimmune disease or cancer had never heard of biosimilars (Jacobs et al., 2016b). Similarly, in a patient survey conducted by the European Federation of Crohn’s & Ulcerative Colitis Associations, 62% of respondents were unfamiliar with biosimilars (Peyrin-Biroulet et al., 2017). Among patients who were familiar with these agents, at least three-quarters expressed concerns, primarily about their safety, efficacy, and molecular basis, and two-thirds would not be fully confident about biosimilars even when prescribed and explained by their clinicians. In a small prospective study of US patients with IBD, 58% of patients were uncomfortable about switching from their current treatment to a biosimilar; 81% and 70% expressed concerns about biosimilar efficacy and safety, respectively, and such concerns were more common in biologic-experienced patients than in biologic-naïve patients (Pineles et al., 2018).

Evidence from a recent Belgian study suggests that clinicians may also lack confidence about biosimilars, as rheumatologists were significantly more likely than patients to express concerns about differences between biosimilars and originator biologics in quality, safety, and price (van Overbeeke et al., 2017). In a survey of US specialty clinicians who frequently prescribed biologic therapies, several important knowledge gaps were identified that could affect clinician–patient communication, including definitions of originator and biosimilar biologics, biosimilar evaluation, and approval processes, and comparability of originator and biosimilar biologics in terms of safety and immunogenicity (Cohen et al., 2017). Interestingly, more than one-third of those participating in the survey believed that biosimilars pose a greater safety risk than originator biologics because of their abbreviated approval process (Cohen et al., 2017). In addition, sizeable minorities of clinicians were uncertain as to whether biosimilars are comparable in efficacy (38%) and safety (43%) to originator biologics (Cohen et al., 2017).

Negative attitudes about biosimilars may be induced in part by information about their lower price. In a study of healthy volunteers who received electrical shocks and rated their pain before and after taking a placebo pill (believed to be a new over-the-counter analgesic), half of the participants were informed that their medication cost $2.50 and the remaining half were informed that their medication was discounted to $0.10, without an explanation for the reduced cost (Waber et al., 2008). Participants who received the expensive medication reported significantly greater pain relief than participants who received the discounted medication, likely because expectations of a correlation between price and efficacy influenced their perception of pain. Tinnermann et al. also found that value information about a medication, such as its cost, can increase the nocebo effect, promoting adverse therapeutic outcomes even in individuals receiving inert treatment for pain (Tinnermann et al., 2017). Specifically, identifying an inactive substance as expensive medication resulted in stronger nocebo hyperalgesia than identifying it as inexpensive medication. In a blinded, randomized study in patients with Parkinson’s disease in which investigators compared the effects of "expensive" placebo with those of "cheap" placebo and levodopa, Espay et al. found that perceived cost was capable of changing brain activation and motor function (Espay et al., 2015).

## Early Evidence: Nocebo Effects With Biosimilars

A lack of awareness, knowledge gaps, and misperceptions about biosimilars may contribute to the development of nocebo effects (i.e., a reduction in treatment benefits) in patients switching from originator biologics to biosimilars (Rezk and Pieper, 2017; Pouillon et al., 2018). Nocebo effects have been proposed in a few clinical studies of biosimilars, including observational studies of infliximab and etanercept originator biologics and their respective biosimilars (Nikiphorou et al., 2015; Tweehuysen et al., 2017; Germain et al., 2018; Scherlinger et al., 2018). In a small, observational, prospective study of patients with established rheumatic disease who consented to switch from originator to biosimilar infliximab (CT-P13), improvements in disease activity and patient-reported outcomes were comparable between therapies 1 year after the transition (Nikiphorou et al., 2015). However, 15% of patients discontinued CT-P13 treatment for subjective reasons, despite having no worsening of disease. The authors of the study suggested that negative expectations of the switch may have played a part in the discontinuations. In a large, observational, prospective cohort study of the effects of switching from originator infliximab to CT-P13 in consenting patients with rheumatic disease, Tweehuysen et al. reported that most patients completed the transition without changes in efficacy, safety, or immunogenicity after 6 months of follow-up (Tweehuysen et al., 2017). Nearly one-quarter of patients nevertheless discontinued CT-P13 therapy during this interval, primarily because of subjective worsening of disease activity and/or tolerability, which the investigators attributed to nocebo effects rather than pharmacological differences between originator and biosimilar agents.

Disease activity was found to be similar before and after patients with rheumatic disease underwent a non-medical switch from originator infliximab to CT-P13 in the DANBIO registry (Glintborg et al., 2017b). However, the adjusted retention rate with CT-P13 after 1 year was significantly lower than that for the historical originator infliximab cohort, suggesting a possible nocebo effect. Similarly, Scherlinger et al. reported lower retention rates after patients with various rheumatic diseases were switched from originator infliximab to CT-P13 compared with control cohorts, but the difference was negligible after patients who discontinued without objective disease activity were excluded (Germain et al., 2018; Scherlinger et al., 2018). In a 1-year pragmatic study of non-medical biosimilar switching in consenting patients with chronic immune-mediated inflammatory disease, including IBD and rheumatic diseases, Boone et al. examined the frequency of nocebo-effect responses, defined as unexplained, negative therapeutic effects occurring after the switch from originator to biosimilar infliximab followed by the return of beneficial effects after re-initiation of originator infliximab (Boone et al., 2018). Although the effectiveness, tolerability, and immunogenicity profiles of originator and biosimilar infliximab were similar, the investigators found an overall nocebo response rate of 13% in patients transitioning from original to biosimilar infliximab, with similar rates in the IBD and rheumatology groups (Boone et al., 2018). The patients presenting with nocebo responses reported "less exerted effect", infusion reactions, and headache, leading investigators to conclude that non-medical switching may have prompted nocebo effects on patients’ disease burden and sense of well-being.

In a recent systematic literature review, Odinet et al. compared efficacy and safety outcomes of a switch from originator biologic to biosimilar products in open-label and double-blind studies to evaluate the possible occurrence of nocebo effects (Odinet et al., 2018). The researchers found higher discontinuation rates for any reason, adverse events, and lack of efficacy in biosimilar infliximab open-label studies versus double-blind studies, suggesting that nocebo effects may inhibit the adoption of this biosimilar. As summarized in **Table 1**, Kristenson et al. noted that authors of five recent studies also proposed a possible nocebo influence when explaining findings in patients switched from an originator biologic to a biosimilar agent (Nikiphorou et al., 2015; Glintborg et al., 2017a; Glintborg et al., 2017b; Tweehuysen et al., 2017; Germain et al., 2018; Scherlinger et al., 2018). However, because relatively few studies have been conducted, current evidence is considered inadequate to confirm such effects.

**Table 1:** A summary of evidence supporting nocebo effects in biosimilar switching studies in patients with rheumatic disease. Adapted from Kristensen LE et al. BioDrugs. 2018 Oct;32(5):397-404 (Kristensen et al., 2018).

Interventions	Study (year)/study design	Evidence
Infliximab/ CT-P13	(Nikiphorou et al., 2015) Observational, single-center	11/39 (28%) patients discontinued CT-P13 6/39 (15%) patients discontinued CT-P13 for subjective reasons No objective worsening of disease activity
	(Glintborg et al., 2017b) Observational registry	117/792 (15%) patients discontinued CT-P13 Main reasons for discontinuation: perceived loss of efficacy [51/792 (6%)] and adverse events [34/792 (4%)] Majority of patients had no change in disease activity
	(Tweehuysen et al., 2017) Observational, multicenter, prospective cohort	44/192 (23%) patients discontinued CT-P13 Main reasons for discontinuation: perceived loss of efficacy [35/192 (8%)] and adverse events [23/192 (12%)] No changes in efficacy, safety, or immunogenicity
	(Scherlinger et al., 2018); (Germain et al., 2018) Observational, single-center	64/89 (72%) patients continued CT-P13 for 33 weeks (median) 25/89 (28%) patients asked to be switched back to originator Reasons for switch back: clinical disease activity [13/25 (52%)]; serum sickness [1/25 (4%)]; no objective disease activity [11/25 (44%)]
Etanercept/ SB4	(Glintborg et al., 2017a) Observational registry (n = 1548)	129/1548 (8%) patients discontinued SB4 during 5-month follow-up Reasons for discontinuation: perceived lack of efficacy [59/1548 (4%)]; adverse events [42/1548 (3%)]; remission [2/1548 (< 1%)]; cancer [4/1548 (< 1%)]; death [1/1548 (< 1%)]; and other/unknown [21/1548 (1%)] Disease activity was unaffected in most patients (3 months pre-switch vs. 3 months post-switch)

Although preliminary, this evidence suggests that nocebo effects associated with switching patients from originator biologics to biosimilars can have unfavorable consequences for patients as well as healthcare systems. Non-adherence to or discontinuation of treatment, and perceived increases in adverse events and suboptimal efficacy, can substantially impair quality of life, lead to higher treatment costs, and damage the patient–clinician relationship (Rezk and Pieper, 2017; Kristensen et al., 2018). The occurrence of potential nocebo responses and their negative impact is also supported by findings from retrospective analyses of randomized controlled trials for anti-migraine medication (Amanzio et al., 2009), antidepressants (Rief et al., 2009), and statins (Rief et al., 2006), in which relatively high rates of adverse events and discontinuation in the placebo arms were likely influenced by patient and investigator expectations. In addition, verbal information about treatment safety/tolerability has also been associated with nocebo responses in prospective investigations. For example, finasteride-treated patients with benign prostatic hyperplasia who were informed about possible sexual dysfunction were significantly more likely to experience sexual adverse effects than those who were not informed (Mondaini et al., 2007). Similar scenarios are anticipated in clinical practice, where patients are counseled by healthcare professionals and have access to treatment information from many possible sources.

## Mitigation of Nocebo Effects on Biosimilar Implementation

Nocebo effects are recognized as negatively affecting adherence to medication, adverse events, and symptom relief during routine treatment (Colloca and Finniss, 2012) and are strongly suspected to similarly influence clinical outcomes in patients switching from originator biologics to biosimilars (Kristensen et al., 2018). Consequently, consideration should be given to ways of minimizing or avoiding these effects. Despite progress in elucidating the mechanisms underlying nocebo effects, as well as many predictive factors, identification of patients who may be particularly vulnerable to nocebo effects is not yet feasible (Colloca, 2017b). However, several clinical and contextual aspects that may be particularly critical to the formation of nocebo effects have been uncovered, including patients’ lack of positive information, negative information provided in print or other materials, negative patient–clinician communication and interaction during treatment, and patients’ emotional burden during treatment (Klinger et al., 2017). Strategies addressing these and other factors that shape patients’ negative expectations may help reduce the likelihood of nocebo effects and improve treatment outcomes (Colloca and Finniss, 2012; Bingel, 2014).

## Education of Patients and Healthcare Professionals

Educational initiatives have been launched by regulatory authorities such as the EMA and FDA to help close gaps in patients’ and healthcare professionals’ awareness and knowledge of biosimilars and their clinical use (European Medicines Agency and European Commission, 2016; European Medicine Agency and European Commission, 2017; US Food and Drug Administration, 2018). Nocebo effects are not specifically mentioned in these materials, but patients are advised to take several steps that may help mitigate nocebo effects: become fully informed about what to expect when switching from an originator to a biosimilar product; obtain information about treatment as needed from healthcare professionals; and be involved in the decision-making about the treatment course.

Professional medical societies such as the European Society for Medical Oncology (Tabernero et al., 2016) and the American Society of Clinical Oncology (Lyman et al., 2018) have published literature on biosimilars and their clinical implementation, which strongly emphasizes the importance of education and open patient–clinician dialogue as a means of ensuring biosimilar acceptance. Recently, an international task force on rheumatologic diseases issued recommendations that will likely reduce potential nocebo effects, addressing the misconception that the lower price of biosimilars connotes lower quality than bio-originators, and emphasizing the importance of patient–healthcare provider consultation in therapeutic decision-making (Kay et al., 2018). In a practical guide for nurses involved in switching patients between similar biological medicines, the European Specialist Nurses Organisation specifically addresses the problem of nocebo effects, providing information on how to respond to patients’ questions about biosimilar cost/quality, how to directly inform them of potential nocebo effects, and how to advise them about avoidance measures (e.g., “trust and belief are important for good efficacy”) (European Specialist Nurses Organisations, 2018).

## Communication and Interaction Between Patients and Healthcare Professionals

**Figure 1:**
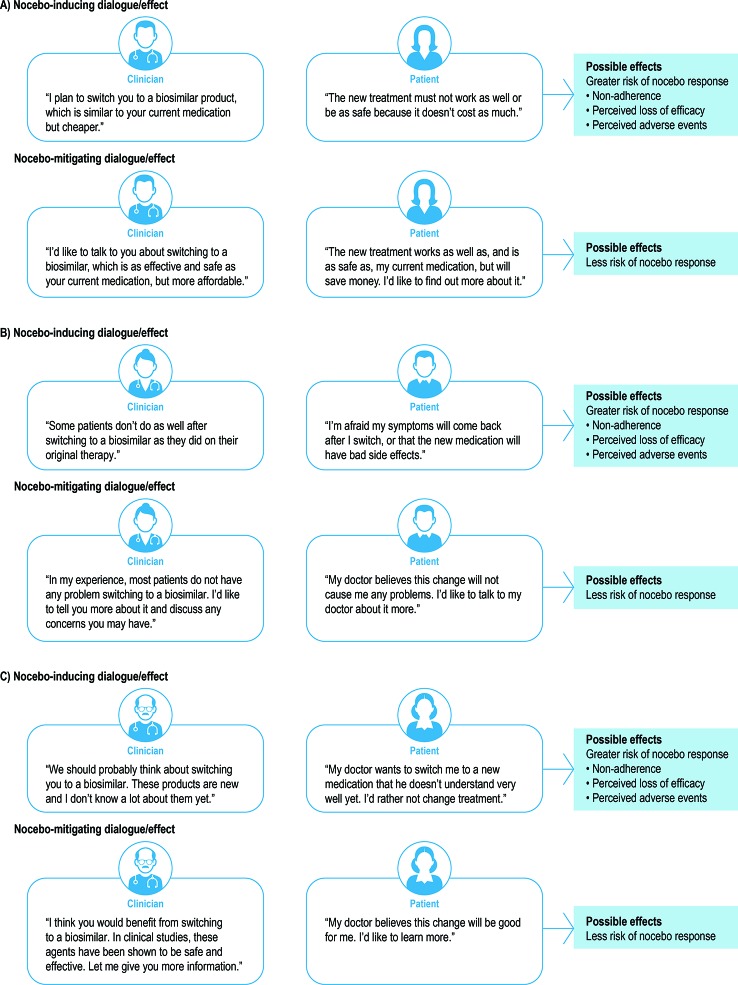
Examples of patient–clinician interaction that may trigger/avoid nocebo effects in patients switching from an originator biologic to a biosimilar.

Managing patients’ beliefs and expectations is an essential strategy in diminishing the risk of nocebo effects. A fine balance must be achieved between, on the one hand, sharing relevant clinical information with patients to promote their autonomy in the decision-making process and, on the other hand, avoiding negative instructions and a negative therapeutic context that could produce negative bias and expectations (Colloca and Finniss, 2012; Hauser et al., 2012). In **Figure 1**, we present several original examples of patient–clinician communication that may encourage or discourage nocebo effects in patients who switch from originator biologic to biosimilar therapy. Healthcare professionals may effectively “frame” information shared with patients by describing treatment benefits and risks in a non-deceptive and reassuring manner, with a focus on positive attributes to help avoid negative expectations (Colloca, 2017b). In sharing information about a proposed treatment, healthcare providers can emphasize that the treatment has been shown to be effective, safe, and well tolerated in the majority of patients, rather than focusing on the minority of patients who may not respond or who experience adverse events (Colloca and Miller, 2011; Colloca, 2017b). Other considerations for clinicians include avoiding negative phrases in the description of treatments; exploring patients’ possible pre-existing beliefs about treatment and/or negative treatment history; paying attention to contextual aspects such as cost; and allowing time for patients to ask questions about negative features of the treatment and to internalize the information shared (Colloca, 2017a; Colloca, 2017b). Healthcare professionals may also choose to educate patients about the possible occurrence of nocebo effects so that they understand the potential psychophysiological mechanisms involved, including anxiety, and the potential detrimental impact.

Using a qualitative research model to explore the impact of non-medical switching from originator to biosimilar products in Danish patients with chronic arthritis, Jørgensen et al. identified several communication strategies that may be essential to avoiding nocebo effects (Jørgensen et al., 2017; The Parker Institute, 2018). When introducing a biosimilar, healthcare providers need to communicate clearly about the products and the switch, using encouraging rather than discouraging language, and establish an open dialogue with patients, allowing adequate time for discussion of relevant educational resources. Systematic education and consistent communication (i.e., “speaking with one voice”) among all healthcare professionals directly involved in helping the patient transition to a biosimilar was also found to be important.

Healthcare professionals’ adoption of a caring, empathetic, and positive attitude may help shape a therapeutic environment that increases placebo effects and reduces nocebo effects in their patients (Data-Franco and Berk, 2013). Verbal and non-verbal communication and behavior influence dialogue and the clinical engagement/relationship (Drossman, 2013). The success of medical interviews/consultations can be influenced by the clinicians’ non-verbal cues in the form of close positioning relative to patients, gentle tone of voice, strong eye contact, and affirmative head nods and gestures (Silverman and Kinnersley, 2010; Drossman, 2013). The importance of reciprocal interactions between patients and clinicians to the understanding of placebo and nocebo responses and treatment outcomes is also underscored by other research findings in the field of pain. Like patients’ treatment expectations, clinicians’ treatment expectations have also been shown to influence placebo analgesia (Gracely et al., 1985). In addition, researchers have demonstrated that clinicians’ administration of pain relief leads to increased brain activation in regions associated with expectancy for pain relief, and their ability to share their patients’ perspective during treatment leads to increased brain activation in regions associated with feelings of reward (Jensen et al., 2014).

Patient-centered care in chronic diseases such as rheumatoid arthritis has been shown to have a positive effect on treatment outcomes, including effectiveness, safety, and costs (Voshaar et al., 2015). This type of care involves patient education, empowerment, involvement, self-management, and shared decision-making. Patients who are encouraged to have a sense of control and ownership of the decision-making process may also be less susceptible to nocebo effects (Data-Franco and Berk, 2013; Voshaar et al., 2015; Boone et al., 2018). In a study of patients with rheumatic diseases and IBD, a strategy based on shared therapeutic decision-making, informed consent, and patient empowerment was implemented to reduce nocebo response rates associated with transitioning to biosimilar therapy (Boone et al., 2018). Although the study was not controlled to evaluate the impact of this strategy, the investigators proposed that patient empowerment likely decreased nocebo response, maintaining the effectiveness and tolerability of the biosimilar after switching.

Importantly, healthcare professionals who discuss the potential use of biosimilars with patients need to be well informed themselves so that they can confidently and reassuringly deliver the necessary information to patients and help them make an informed, shared decision without triggering negative expectations and potential nocebo responses (Colloca and Finniss, 2012; Chavarria et al., 2017; Rezk and Pieper, 2017). When discussing biosimilar use with patients, clinicians may offer only the relevant details, including the definition of biosimilars and information about the evidence/clinical trials required for biosimilar approval and similarities between originator biologics and biosimilars (**Table 2**) (Jacobs et al., 2016b; Jorgensen et al., 2017; Rezk and Pieper, 2017; Cohen et al., 2018; Zhao et al., 2018).

**Table 2:** Questions about biologic/biosimilar therapy for healthcare professionals to answer when discussing biosimilar use with patients (Jacobs et al., 2016b).

Type	Question
Background	How are biologics used in the patient’s specific type of chronic immune-inflammatory disease? How are biosimilars defined?
Evidence	What evidence is needed for a biosimilar to be approved? What clinical trials are conducted to evaluate biosimilars?
Characteristics	Are biosimilars similar to originator biologics in efficacy? Are biosimilars similar to originator biologics in safety? How will the biosimilar be administered?
Practical/logistical information	Does the patient have access to biosimilar treatment? What will insurance coverage/out-of-pocket costs be? What services will be available to support the patient? Which company manufactures the biosimilar?

## Evidence of Mitigation Strategy Effectiveness

Although additional research is needed, findings of recent investigations suggest that mitigation strategies may be effective against nocebo effects (Bartels et al., 2017; Tweehuysen et al., 2017). In a randomized controlled study using electrical and histaminic itch stimuli, Bartels et al. demonstrated that induction of positive expectations by conditioning with verbal suggestion can minimize and even reverse nocebo effects (Bartels et al., 2017). Moreover, in the switching study conducted by Tweehuysen et al., an enhanced communication strategy may have increased acceptance and persistence rates among patients with rheumatic disease after they transitioned from an originator biologic to a biosimilar (Tweehuysen et al., 2017). Communications were increased for patients who consented to switch from originator etanercept to biosimilar SB4, but not for patients who consented to switch from originator infliximab to biosimilar CT-P13. The enhanced communications strategy included informing all patients directly and simultaneously (followed by a national media item); reporting the reasons for transitioning (i.e., lower cost and fewer injection-site reactions based on findings of a previous clinical trial); and “soft skills” training for rheumatology and pharmacy staff, including on the nocebo concept. Over 6 months of follow-up after switching, 6% of patients who received enhanced communications (i.e., those in the originator etanercept/SB4 group) discontinued biosimilar treatment compared with 24% of patients who did not receive enhanced communications (i.e., those in the originator infliximab/CT-P13 group). In addition, the absence of group-think effects may have improved acceptance/persistence rates after switching. Patients in the enhanced communications group, who were less likely to discontinue after transitioning to biosimilar therapy, received treatment *via* individual subcutaneous administration, whereas those in the no-enhanced communications group, who were more likely to discontinue after transitioning, received treatment *via* intravenous infusion in a group setting.

## Limitations and Strengths

As described, this is a qualitative, scoping review and may not capture all the body of the available literature on nocebo effects and biosimilars. Despite this limitation, the strengths of this review are that we covered several perspectives and angles including knowledge gaps, misperceptions, and negative expectations on biosimilar therapy and nocebo effects. We discussed evidence of nocebo effects with some of the biosimilars and commented on some possible strategies to overcome nocebo effects with an emphasis on the importance of education, and effective communication among patients, healthcare professionals and families.

## Conclusions

Nocebo effects represent new or worsening symptoms or adverse events that occur largely as a consequence of patients’ negative expectations rather than *via* a mechanism of action of the treatment itself. Like their placebo counterparts, nocebo effects are a consequence of the interaction among myriad psychosocial, neurobiological, and contextual/environmental factors. Although rigorous studies are conducted to demonstrate the comparable quality, safety, and efficacy of biosimilars vis-à-vis biologics before their regulatory approval, gaps in patients’ and healthcare professionals’ awareness, understanding, and perception of biosimilars may contribute to nocebo effects and diminish their clinical benefits.

As biosimilars will continue to play a critical role in expanding treatment options and access to safe and effective biologic therapies, adoption of nocebo-reducing strategies is crucial, including avoidance of negative instructions and expectations, framing information to focus on positive attributes, and promotion of shared decision-making and patient empowerment. Healthcare professionals who adopt a warm and empathetic attitude, and are fully confident in their knowledge of biosimilars and awareness of bias-inducing factors, may help minimize the risk of nocebo effects and improve patients’ adherence to these agents. Although not specifically addressed in this review, health authorities and health insurance companies will likely play a critical role in supporting further discussion and education of clinicians and patients about the implications of nocebo phenomena and biosimilars to enhance the acceptance and utilization of these agents. However, additional research is needed to support existing strategies and develop novel approaches that will further reduce negative expectations and improve the psychosocial and environmental context contributing to nocebo effects with biosimilar implementation.

## Author Contributions

LC, RP, and TM reviewed the literature and contributed to the drafting of the manuscript. All authors also edited, revised, and approved the final version of the review.

## Funding

This work was funded by Pfizer Inc.

## Conflict of Interest

TM was an employee of Pfizer Inc and owned Pfizer stock at the time of this manuscript’s submission. 

The remaining authors declare that the research was conducted in the absence of any commercial or financial relationships that could be construed as a potential conflict of interest.
